# Niche–trait relationships at individual and population level in three co‐occurring passerine species

**DOI:** 10.1002/ece3.7569

**Published:** 2021-05-02

**Authors:** Pei‐Jen L. Shaner, Yin‐Kai Chen, Yu‐Cheng Hsu

**Affiliations:** ^1^ Department of Life Science National Taiwan Normal University Taipei Taiwan; ^2^ Department of Natural Resources and Environmental Studies National Dong Hwa University Hualien Taiwan

**Keywords:** beak, diet, foraging, functional traits, phenotypic variation, trophic ecology

## Abstract

The niche variation hypothesis (NVH) predicts that populations with wider niches exhibit greater morphological variation through increased interindividual differences in both niche and morphology. In this study, we examined niche–trait relationships in three passerine species (*Cyanoderma ruficeps*, *Sinosuthora webbiana*, and *Zosterops* *simplex*). A total of 289 *C. ruficeps* from 7 sites, 259 *S. webbiana* from 8 sites, and 144 *Z. simplex* from 6 sites were sampled along an elevation gradient (0–2,700 m) in Taiwan from 2009 to 2017. We measured bill traits (length, width, and depth of bill) and body size traits (length of head, tarsus, and wing) of the birds, which were reduced to four principal components (bill PC1, bill PC2, body size PC1, and body size PC2). We collected feather tissues for stable carbon and nitrogen isotope analyses to quantify their isotope niche. We quantified interindividual differences in isotope space and trait space with four diversity metrics (divergence, dispersion, evenness, and uniqueness) and tested whether interindividual differences in isotope space and trait space are positively associated. We quantified population isotope niche width by Bayesian ellipse area and population morphological variation by variances of the PCs. The results showed that individual uniqueness in isotope niche and bill morphology (average closeness of individuals within the population isotope/trait space) were positively associated across three species. Furthermore, isotope niche width and bill PC1 (reflecting the size of bill) variation at population level were also positively associated across the three species, supporting the NVH. Of the three species, *C. ruficeps* and *S. webbiana* showed stronger support for the NVH than *Z. simplex*, possibly due to the latter having narrower elevational distribution and a more specialized, plant‐based diet. The diversity metrics represented different aspects of interindividual differences in niche/trait space, and for the passerines, individual uniqueness appeared to play an important role in their niche–trait dynamics.

## Graphical abstract

This study provided empirical evidence for the niche variation hypothesis in three passerine species (*Cyanoderma ruficeps*, *Sinosuthora webbiana*, and *Zosterops* *simplex*) in Taiwan. All three species showed positive associations between isotope niche width and bill size variation. Furthermore, all three species shared the same pattern that as individual birds occupied unique bill trait space within the population, they also tended to occupy unique isotope space, suggesting niche width expansion at population level through morphological differentiation at individual level.

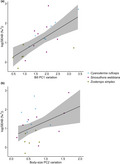

## 1 INTRODUCTION

Trait‐based research is a powerful approach to explore complex eco‐evolutionary dynamics (e.g., Adler et al., 2013; Foden et al., 2013; Luck et al., 2012; Pigot et al., 2020). Morphological traits hold great potentials for trait‐based ecology because these data are readily available for a large number of taxa from field measurements, museum specimen, and fossil records. However, morphological traits could serve multiple functions and influence various niche dimensions, and vice versa, weakening the covariance among morphological traits, ecological functions, and niche dimensions (e.g., Felice et al., 2019; Kennedy et al., 2020; Navalón et al., 2019). Such “many‐to‐one” or “one‐to‐many” relationships between morphological traits and ecological functions both create challenges and offer opportunities for trait‐based and niche‐based predictions (Kennedy et al., 2020; Reif et al., 2016; Wainwright et al., 2005; Webb et al., 2010).

The niche variation hypothesis (NVH; Van Valen, 1965) predicts that populations with broader niche widths should exhibit greater individual specialization (IS) in niche use (Bolnick et al., 2003). Consequently, for morphological traits that are closely linked to niche use at individual level, the NVH also predicts that populations with broader niche widths should exhibit greater morphological variation as interindividual differences within the population's niche space and trait space increased concurrently. The NVH has found some empirical support from a range of animal taxa including fish, birds, reptiles, gastropods, and insects (e.g., Bolnick et al., 2007 and references therein; Cloyed & Eason, 2017; Costa et al., 2008; Hsu et al., 2014; Maldonado et al., 2017; Santoro et al., 2011). Previous studies primarily focused on demonstrating a positive correlation between niche width and IS, or between niche width and morphological variation. However, the pattern of interindividual differences within the population's niche/trait space, which underlies the NVH, is also an important component of the niche–trait dynamics but remains little studied.

Species may differ in their strength of the NVH and the associated niche–trait relationships depending on their evolutionary histories and ecological circumstances. For example, Cloyed and Eason (2017) found evidence for the NVH (IS in diet increased with population diet niche width) in two of the five anuran species studied. They suggest that the species difference might be related to the level of morphological constraints on feeding. Namely, the two species exhibiting the NVH had intermediate gape width to body length ratios (gape width constrains prey size in anurans), which allows for the morphological space that individuals need to shift their diets to when necessary. While this study directly measured degree of IS with dietary data rather than using gape width as a proxy, it illustrates the point that across the range of trait values for an assemblage, species occupying intermediate range of trait values may have higher tendencies to exhibit the NVH. Another example by Maldonado et al. (2017) reported evidence for the NVH across 12 passerine species in a single community. They found that omnivores tended to have higher IS than insectivores, suggesting that species occupying intermediate trophic positions are the ones whose individuals can have the niche space they need to shift their diets to when necessary. Therefore, species occupying intermediate trait and niche position in a community may be more likely to exhibit the NVH. By studying co‐occurring species in and across communities, we could gain insights into the evolutionary and ecological factors that help generate species‐specific patterns in niche–trait relationships.

Birds as a group have been extensively studied with respect to their morphological traits (Kennedy et al., 2020; Pigot et al., 2016, 2020; Ricklefs, 2004, 2012). Passerines are the most diversified avian lineage, and variation in their morphological, ecological, and life‐history traits has offered great insights into the processes of adaptive divergence (Boag & Grant, 1981; McCormack & Smith, 2008), community structure (Pigot et al., 2016; Ricklefs, 2012), and biological responses to environmental forcing (Devictor et al., 2007; Gardner et al., 2009). Morphological traits of passerines are associated with many aspects of their ecology, including diet and foraging (Benkman, 1993; Botero‐Delgadillo & Bayly, 2012; Leisler et al., 1989; Marchetti et al., 1995; Miles & Ricklefs, 1984; Pigot et al., 2016; Schluter & Grant, 1984), habitat and substrate use (Babbington et al., 2020; Leisler et al., 1989; Suhonen et al., 1994), flight and locomotion (Leisler et al., 1989; Norberg, 1979), climate and thermoregulation (Allen, 1877; Bergmann, 1847; Greenberg et al., 2012; Greenberg et al., 2012; Symonds & Tattersall, 2010; Tattersall et al., 2017), and vocalization (Giraudeau et al., 2014; Greenberg & Olsen, 2010; Podos & Nowicki, 2004).

Previous studies suggest that morphological traits in birds are generally linked to their trophic ecology (e.g., Miles & Ricklefs, 1984; Pigot et al., 2016, 2020; Quiroga et al., 2018). For example, using nine morphological traits for 9,963 bird species, Pigot et al. (2020) showed that the position of a species in morphological space can be reliably mapped to trophic niche axes. Furthermore, Pigot et al. (2016) demonstrated that morphological traits of 523 passerine species captured more than half of the variation in species trophic niches. Passerines have broad diet niche spanning across multiple trophic levels (Ramirez‐Otarola et al., 2011), and their morphological traits have been shown to correlate with their trophic ecology. For example, slender bills, shorter tarsus length, and smaller body size have been associated with insectivorous diets (Hsu et al., 2014; Quiroga et al., 2018). Specifically, slender bills allow birds to pick and probe insects efficiently, shorter tarsus allows birds to use tree trunk as foraging substrate, and smaller body size precludes large prey such as vertebrates (Quiroga et al., 2018). Therefore, passerines are excellent candidates for studying the NVH and the associated niche–trait relationships.

For large‐scaled studies involving hundreds of species, using species‐level guild assignment to quantify trophic niche is reasonable and effective (Wilman et al., 2014). However, at individual, population, and community levels, more quantitative measures of the birds' trophic niche are often required. Stable carbon and nitrogen isotopes (δ^13^C, δ^15^N) are increasingly used to quantifying organisms' trophic positions, as well as interindividual differences in trophic niche, population niche width, and species niche overlap in a community (Bearhop et al., 2004; Cucherousset & Villéger, 2015; Jackson et al., 2011; Layman et al., 2007; Yeakel et al., 2016). Recently, stable isotopes have been successfully applied to passerines to quantify their trophic niche and test the NVH (e.g., Hsu et al., 2014; Maldonado et al., 2017). Although stable carbon and nitrogen isotope values can vary from site to site due to biogeochemical processes and anthropogenic influences (O'Leary, 1988; Wang et al., 2007), it is possible to adjust for site‐specific baseline (e.g., using plants' foliar isotope values in terrestrial ecosystems as baseline) and make meaningful comparisons on organisms' trophic niche across sites. When using isotope values to quantify niche, however, the term “isotope niche” is more appropriate than “trophic niche” because (a) isotope values only constitute parts of an organism's trophic niche as they may not reflect foraging behaviors and substrate use; (b) isotope values can also reflect habitat and vegetation characteristics (plant isotope values can vary with environmental conditions such as light, soil nitrogen, mycorrhizal association, and water stress; e.g., Marshall et al., 2007; Wang et al., 2007; Zheng & Shangguan, 2007).

In multidimensional niche/trait space, the geometric arrangement of individuals within a population provides useful metrics for quantifying interindividual differences. For example, Cucherousset and Villéger (2015) proposed four metrics to quantify isotope niche diversity, which were adapted from existing metrics for functional trait diversity (Villéger et al., 2008). Therefore, they can be applied to both isotope niche and morphological traits. The four metrics are as follows: (a) divergence, which measures the degree to which the individuals are close to the population center (it tends to 0 when most of the points are close to the center of the isotope/trait space, and it approaches 1 when most of the points are located on the edges of the isotope/trait space); (b) dispersion, which measures the deviation the individuals are to the center of the population divided by the maximal distance to the center (it tends to 0 when most of the points have the same isotope/trait values, and it increases when most of the points have contrasted isotope/trait values); (c) evenness, which measures the regularity in the distribution of individuals along the shortest tree that links all the individuals in the population (it tends to 0 when most of the points are packed within a small region of the isotope/trait space, and it approaches to 1 when most of the points are evenly distributed in the isotope/trait space); (d) uniqueness, the inverse of the average isotopic/trait redundancy which reflects the average closeness of individuals within the isotope/trait space (it tends to 0 when each point has at least one point with the same position within the isotope/trait space, and it approaches 1 when most of the points are isolated in the isotope/trait space). All four metrics increase in value when interindividual differences in isotope niche space and morphological trait space increase. Therefore, a positive relationship between isotopic diversity and trait diversity across populations could facilitate the NVH.

In this study, we examined the relationship between isotope niche and morphological trait and tested the NVH in three passerine species, rufous‐capped babbler *Cyanoderma ruficeps*, vinous‐throated parrotbill *Sinosuthora webbiana*, and Swinhoe's white‐eye *Zosterops* *simplex*. All three species are small‐sized passerines widely distributed in eastern Asia and frequently co‐occurring in various habitats such as forests and shrublands (Robson, 2007; Severinghaus et al., 2012). In Taiwan, *S. webbiana* have the widest elevational range (0–3,100 m; Robson, 2007), followed by *C. ruficeps* (0–2,500 m; Yen, 1990) and *Z. simplex* (0–1,200 m; Severinghaus et al., 2012). Although all three species are omnivorous, observational studies suggest that *Z. simplex* (feeding on insects, fruits, and nectars; Chen & Chou, 1999; Wilman et al., 2014) may be more specialized than *C. ruficeps* and *S. webbiana* (feeding on insects, fruits, seeds, flowers; Chen & Chou, 1999; Severinghaus et al., 2012). On the other hand, *C. ruficeps* may be more restricted in habitat use (forests and shrubs; Myers, 2009) than *S. webbiana* (shrubs, woodlands, forest edges, wetlands; Robson, 2007) and *Z. simplex* (shrubs, woodlands, anthropogenic environments such as parks, school campuses, and orchards; Severinghaus et al., 2012). At microhabitat scale, *C. ruficeps* are known to be highly sedentary, foraging mostly in the understory (Myers, 2009). While *S. webbiana* also prefer understory and avoid flying across open areas by zigzagging through branches (Severinghaus, 1991), they do use canopy when there are abundant plant foods available (Severinghaus, 1991). Compared to *C. ruficeps* and *S. webbiana*, *Z. simplex* are more conspicuous and frequently use canopy (Y. Hsu, personal observation).

Of the three species, we expect *S. webbiana* to have a more flexible isotope niche than *C. ruficeps* and *Z. simplex*. This is because *S. webbiana* have wide elevational range, broad diet, and generalized habitat use. A previous study had shown that *S. webbiana* exhibited increased bill morphological variation with increasing isotope niche width, supporting the NVH (Hsu et al., 2014). Between *C. ruficeps* and *Z. simplex*, however, it is more difficult to predict which species would have a more flexible isotope niche. On the one hand, *C. ruficeps* may have a broader diet; on the other hand, *Z. simplex* tend to use more varied habitats, microhabitats, and substrates. Given the ecological differences among the three species, *S. webbiana* may exhibit stronger niche–trait relationships than *C. ruficeps* and *Z. simplex*. Here we asked three specific questions: (a) Are interindividual differences in isotope niche space positively associated with that in morphological trait space? (b) Does population morphological variation increase with isotope niche width (NVH)? (c) Are there species‐specific patterns? In particular, do *S. webbiana* exhibit stronger niche–trait relationships than *C. ruficeps* and *Z. simplex*?

## 2 MATERIALS AND METHODS

### 2.1 Study system and sampling

The study included 10 sites along an elevational gradient from 0 to 2,700 m in eastern Taiwan (Table 1 and Figure S1). The distances between sites are 2–24 km. All 10 sites are composed of mosaics of forests and open vegetations such as bushes, grasslands, abandoned/fallow farmlands. The lower elevation sites (DON, CHO, SAZ, ZHA) are in rural setting, whereas the higher elevation sites (XIB, LIA, LUS, CIE, GUA, HEH) are within the recreational area of a national park (Taroko National Park). Therefore, all sites receive low to intermediate anthropogenic influences. However, none of them are in urban area or pristine forests. Given that *S. webbiana* have a relatively small home range (<1 km; Lee et al., 2010) and there was no cross‐site recapture for *C. ruficeps* and *Z. simplex* (Y. Hsu, unpublished data), we treated each site as a population in this study.

**TABLE 1:** Study sites and sample sizes. The sampling was done between 2009 and 2017

Site	Location	Elevation (m)	Habitat and vegetation description	Species richness	Sample size (female, male)		
					*C. ruficeps*	*S. webbiana*	*Z. simplex*
Chongde (CHO)	121°39′37″E; 24°08′54″N	28	Costal bushes	12.8 (52)	38 (15, 23)	44 (22, 22)	18 (10, 8)
Dong Hwa University (DON)	121°32′47″E; 23°56′59″N	41	Grasslands on a rural campus	12.7 (41)	—	40 (13, 27)	6 (3, 3)
Sanzhan (SAZ)	121°37′08″E; 24°06′01″N	61	Costal bushes	10.6 (12)	—	12 (5, 7)	15 (8, 7)
Zhao Feng Farm (ZHA)	121°27′59″E; 23°47′18″N	102	Abandoned/fallow farmland mixed with bushes	9.4 (11)	—	24 (7, 17)	—
Xibao (XIB)	121°28′55″E; 24°12′17″N	967	Evergreen broadleaf forest, mixed with abandoned/fallow farmland and bushes	15.8 (63)	37 (15, 22)	25 (11, 14)	31 (15, 16)
Lianhua Pond (LIA)	121°29′48″E; 24°13′02″N	1,142	Evergreen broadleaf forest, mixed with abandoned/fallow farmland and bushes	11.2 (53)	72 (32, 40)	40 (20, 20)	30 (15, 15)
Luoshao (LUS)	121°27′03″E; 24°12′24″N	1,236	Evergreen broadleaf forest, mixed with abandoned/fallow farmland and bushes	12.5 (75)	60 (20, 40)	58 (27, 31)	44 (25, 19)
Ci′en (CIE)	121°23′18″E; 24°11′31″N	1,986	Evergreen conifer–broadleaf forest, mixed with abandoned/fallow farmland and bushes	12.2 (22)	31 (13, 18)	—	—
Guanyuan (GUA)	121°20′32″E; 24°11′11″N	2,400	Evergreen conifer–broadleaf forest, mixed with abandoned/fallow farmland and bushes	6.0 (6)	10 (5, 5)	—	—
Hehuan Farm (HEH)	121°17′56″E; 24°10′14″N	2,668	Evergreen conifer–broadleaf forest, mixed with abandoned/fallow farmland and bushes	14.4 (44)	41 (16, 25)	16 (11, 5)	—

Note

The sample size represents the number of unique individuals of *Cyanoderma ruficeps*, *Sinosuthora webbiana*, and *Zosterops* *simplex*. Not all species occurred at all sites. Species richness is the rarefied number of bird species captured given the differential sampling efforts across sites (R package “vegan”); actual observed species richness is in parenthesis. Photographs of the study sites taken at the bird‐netting locations are in Figure S1.

Bird netting was performed between 2009 and 2017. Ten to 15 mist‐nets were set up at a site on the day of sampling and checked every 15 min. All three species can be found in forests. However, due to logistic difficulties in setting up mist‐nets in dense vegetation, we conducted the netting in tall grasses or bushes along forest edges. We observed that the netting sites were used as foraging habitats for all three species. Each captured bird was banded for individual identification, and recaptured individuals were excluded from this study. Upon first capture, morphological traits of the birds were measured, and a few feathers from their chest were collected for stable isotope analysis. Approximately 20 µl of blood was obtained by venipuncture from the brachial vein and stored in 100% ethanol for molecular sexing. All individuals were released on site immediately after sampling.

The netting, handling, and sampling procedures were approved by the Institutional Animal Care and Use Committee of National Dong Hwa University, Taroko National Park, Hualien county government, and Taiwan's Council of Agriculture.

### 2.2 Morphological measurement and molecular sexing

A digital caliper (Mitutoyo) and a ruler with a zero‐stop were used to measure six morphological traits (Eck et al., 2012; Figure S2): bill length, bill width, bill depth (bill traits), head length, tarsus length, and wing length (body size traits; Freeman & Jackson, 1990; Gosler et al., 1998; Hamilton, 1961; James, 1970). The measurements were made to the nearest 0.01 mm except for wing length which was made to the nearest 0.1 mm. A total of 36 technicians performed morphological measurements over the years but 84%–93% of each of the three species were measured by the same four technicians. We had considered using only the data measured by these four technicians in preliminary analyses. However, such data treatment would reduce the sample size for one of the bird populations (*Z. simplex* at site DON) to three, which is inappropriate for variation estimates. Alternatively, we excluded the data measured by the technicians with less than three measurement records and then standardized the morphological measurements by controlling the technician effect (see *Statistical analysis*; Tables S1 and S2).

Genomic DNA from blood was extracted using the methods in Gemmell and Akiyama (1996). Fragments of chromo‐helicase‐DNA binding protein (CHD) gene from the sex chromosomes were amplified by polymerase chain reaction (PCR), using the primers 2550F and 2718R (Fridolfsson & Ellegren, 1999). The PCR protocol followed Fridolfsson and Ellegren (1999) with slight modifications (Yang et al., 2012).

### 2.3 Stable isotope analysis

The feather samples were lipid‐extracted in 2:1 chloroform:methanol solution for 24 hr, rinsed with distilled water, and oven‐dried at 55°C for 48–72 hr. Dried feather samples were carefully removed of shafts with surgical forceps and scissors, and approximately 1 mg of the feather tissues were loaded into tin capsules for isotope analysis. In order to provide site‐specific baselines to correct feather isotope values, we collected 8–14 foliar samples of the common plant species at each site (one foliar sample from each plant species; 8–14 plant species across sites) opportunistically in 2009, 2010, 2018, and 2019. The plant foliar samples were rinsed with distilled water, oven‐dried at 55°C for 48–72 hr, and grounded into fine powder. Approximately 3 mg of plant foliar samples were loaded into tin capsules for isotope analysis. Because lipids tend to have more negative carbon isotope values than proteins and carbohydrates, lipid‐extraction for consumer tissues could help reduce the influence of different lipid contents among organisms on their carbon isotope values. On the other hand, all components of plant foliar tissues, including lipids, proteins, and carbohydrates, are eaten by consumers. Therefore, it is not necessary to lipid extract plant samples. Stable carbon and nitrogen isotope analysis was performed at UC Davis Stable Isotope Facility (Thermo Finnigan Delta Plus). The site‐specific baseline (mean isotope values of the plant species at each site) was subtracted from feather values of the birds captured at the site to obtain adjusted bird isotope values (δ^13^C_adj_ = δ^13^C_feather_ − δ^13^C_plant‐foliar_; δ^15^N_adj_ = δ^15^N_feather_ − δ^15^N_plant‐foliar_; for raw feather values and plant foliar values, see Figure S3).

### 2.4 Statistical analysis

#### 2.4.1 Data sets

There are published data available on isotope values and morphological measurements for *S. webbiana* in this study system. We incorporated the data (Hsu et al., 2014) and added new data on additional *S. webbiana* individuals (170 individuals from Hsu et al., 2014 with 89 new individuals from this study, approximately 52% increase in sample size). The sample sizes are 289 for *C. ruficeps* across 7 sites, 259 for *S. webbiana* across 8 sites, and 144 for *Z. simplex* across 6 sites, for a total of 692 individual birds from 21 bird populations (a population comprises the individuals of a given species at a given site; Table 1).

#### 2.4.2 Controlling the technician effect on morphological measurements

To account for the technician effect, we fitted a general linear model to the measurement values of each morphological trait for each species, using technician identity as the fixed effect (Table S1). We controlled technician effect by species because in our preliminary assessments; there was significant species ×technician effect on raw values of all morphological measurements; Table S2). The residuals of the morphological trait, pooled across species, were then entered into principal component analysis (PCA) for bill traits (bill length, bill width, bill depth) and body size traits (tarsus length, wing length, head length), respectively. The first two principal components (PCs) explained an accumulated 82% and 80% of total variance for bill PCA and body size PCA, respectively, which were retained (bill PC1, bill PC2, body size PC1, body size PC2).

#### 2.4.3 Interindividual differences in isotope space and trait space

We calculated four metrics to quantify different aspects of interindividual differences in isotope/trait space following Cucherousset and Villéger (2015). Prior to calculating isotopic divergence, dispersion, evenness, and uniqueness, the baseline‐adjusted isotope values (δ^13^C_adj_ and δ^15^N_adj_) of bird feathers were first scaled to be between 0 and 1 for each population. These scaled isotope values indicate individuals' relative niche positions within a population. Similarly, the trait divergence, dispersion, evenness, and uniqueness were calculated for bill trait (based on scaled bill PC1 and bill PC2 scores) and body size trait (based on scaled body size PC1 and body size PC2), respectively. General linear mixed models (GLMM, R package “lmerTest”) were first fitted to the data with “site” as the random intercept. However, because the “site” effect was close to zero for all metrics, we refitted general linear models (GLM) to the data. Each of the isotopic metrics was the response variable; the same metrics for bill and body size traits, species, as well as two‐way interactions between species and trait metrics, were the fixed effects (higher‐level interactions were not tested given the modest sample size). The initial models were further reduced through model selection to retain only the significant effects, which was followed by post hoc comparisons (Bonferroni adjustment for type I error) to test whether any of the specific‐specific patterns is significant.

#### 2.4.4 Niche variation hypothesis

We quantified population niche width with standardized Bayesian ellipse area (SEAB, R package “SIAR”; Jackson et al., 2011). We used the variations of PC scores (variations of bill PC1 scores, bill PC2 scores, body size PC1 scores, and body size PC2 scores) to reflect morphological variations. We fitted the GLMM (R package “lmerTest”) to the data with “site” as the random intercept. The log‐transformed isotope niche width (log[SEAB]) was the response variable; the variations of the four morphological PCs, species and two‐way interactions between species and the PCs, were the fixed effects (higher‐level interactions were not tested given the modest sample size). The initial models were further reduced through model selection to retain only the significant effects. The significances of the main effects were determined using *F* test with type III analysis based on Satterthwaite's method. Post hoc comparisons were not performed as “species” effect was dropped during model selection, indicating no species‐specific patterns.

## 3 RESULTS

### 3.1 Bill and body size traits

Higher bill PC1 scores indicated larger bill size (longer, wider, and thicker bills), and higher bill PC2 scores slender bills (long and narrow bills; Table 2). Higher body size PC1 scores indicated larger body size (longer heads, tarsus, and wings), and higher body size PC2 scores (longer tarsus with shorter wings; Table 2).

**TABLE 2:** Principal component loadings for bill and body size traits of the passerines

Trait (mm)	Bill PC1	BillPC2	Body size PC1	Body size PC2
Bill length (resids)	0.5765	0.5836	—	—
Bill width (resids)	0.5411	−0.7971	—	—
Bill depth (resids)	0.6122	0.1549	—	—
Head length (resids)	—	—	0.6190	0.0160
Tarsus length (resids)	—	—	0.5576	0.6952
Wing length (resids)	—	—	0.5531	−0.7187

Note

The trait values were the residuals from the linear models that accounted for the technician effect (Table S1). The data on *Cyanoderma ruficeps*, *Sinosuthora webbiana*, and *Zosterops* *simplex* are pooled for the principal component analysis.

Either based on the residuals of the trait values or PC scores, the three species shared similar morphologies (Figures S4 and S5). However, morphological variability in *C. ruficeps* and *S. webbiana* tended to be larger than that in *Z. simplex* (Figures S4 and S5).

### 3.2 Isotope niche

**FIGURE 1:**
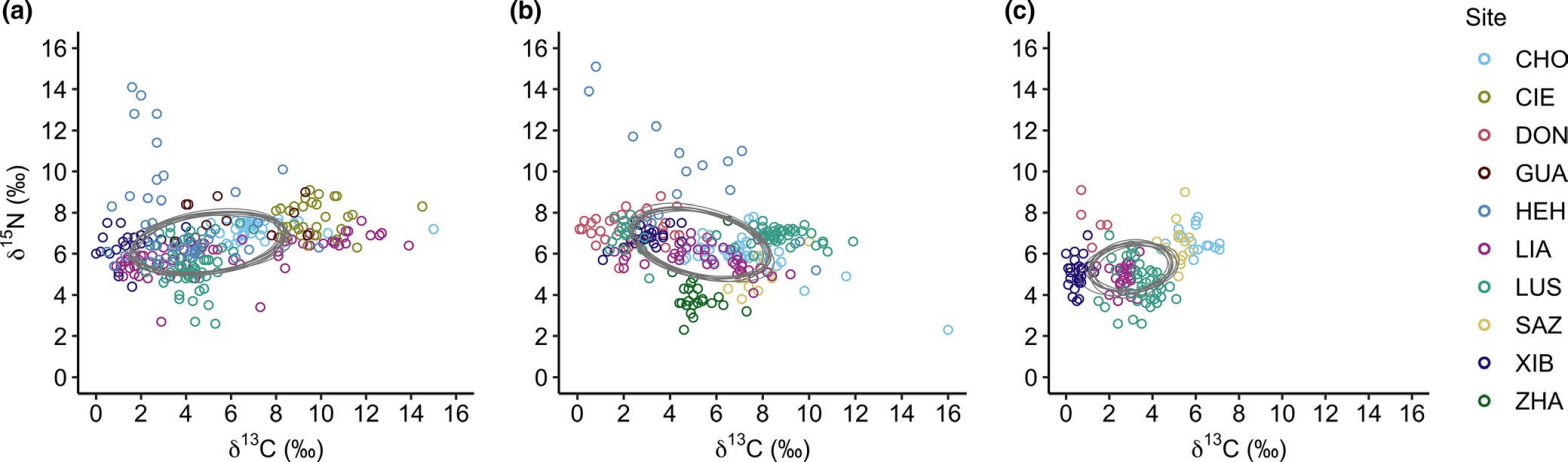
Species‐level isotope niche of the passerines. The niche widths of *Cyanoderma ruficeps* (a), *Sinosuthora webbiana* (b), and *Zosterops* *simplex* (c) were estimated with adjusted isotope values (δ^13^C_adj_ and δ^15^N_adj_) of all individuals pooled across sites. Each unfilled circle represents the isotope values of a unique individual, and the ellipses are 10 posterior draws of the 4,000 Bayesian standard ellipses to illustrate estimated species‐level niche width. Although the sites were pooled, they were colored differently to facilitate visual inspection of the relative positions of different populations (sites)

**FIGURE 2:**
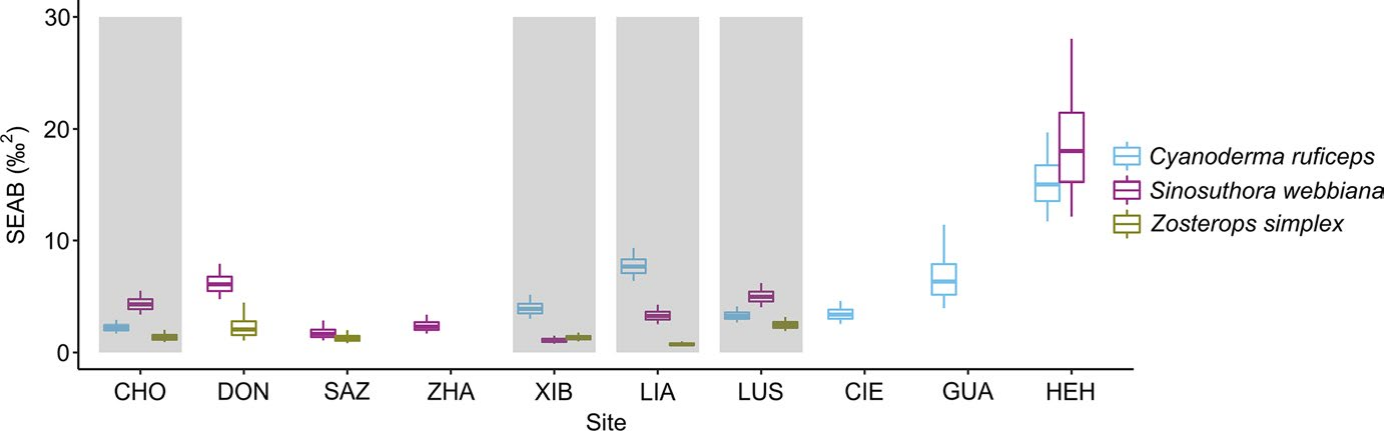
Population‐level isotope niche width of the passerines. The niche widths of *Cyanoderma ruficeps*, *Sinosuthora webbiana*, and *Zosterops* *simplex* were estimated with adjusted isotope values (δ^13^C_adj_ and δ^15^N_adj_) of all individuals within a site. Each boxplot shows the median (the horizontal line), 25th–75th (the box), and 2.5th–97.5th (the whiskers) percentiles of the 4,000 Bayesian standard ellipse areas. The four sites where all three species occurred, CHO, XIB, LIA, and LUS, are highlighted with gray background

The three species occupied a similar isotope niche position (Figure 1 and Figure S3), but *C. ruficeps* and *S. webbiana* had larger species‐level niche widths than *Z. simplex* (the 2.5th–97.5th percentiles of SEAB: *C. ruficeps* = 13.76–16.69‰^2^; *S. webbiana* = 13.05–16.08‰^2^; *Z. simplex* = 6.24–8.17‰^2^). At population level, the three species exhibited dynamic patterns in their niche, varying from site to site (Figure 2 and Figure S6). Of the four sites where all three species coexisted, *C. ruficeps* and *S. webbiana* each had the largest niche width at two sites, whereas *Z. simplex* consistently had the smaller niche width. Across the sites for a given species, population niche width (median SEAB) ranged 2.22–14.98‰^2^, 1.09–17.81‰^2^, 0.74–2.46‰^2^ for *C. ruficeps*, *S. webbiana*, and *Z. simplex,* respectively. Therefore, at both species and population level, *C. ruficeps* and *S. webbiana* had larger niche width than *Z. simplex*. For the site at the highest elevation (HEH), *C. ruficeps* and *S. webbiana* both exhibited very large population niche width, which greatly expanded their species niche width. By contrast, *Z. simplex* was absent from this high‐elevation site, which might have contributed to their smaller species‐level niche width.

### 3.3 Interindividual differences in isotope space and trait space

**FIGURE 3:**
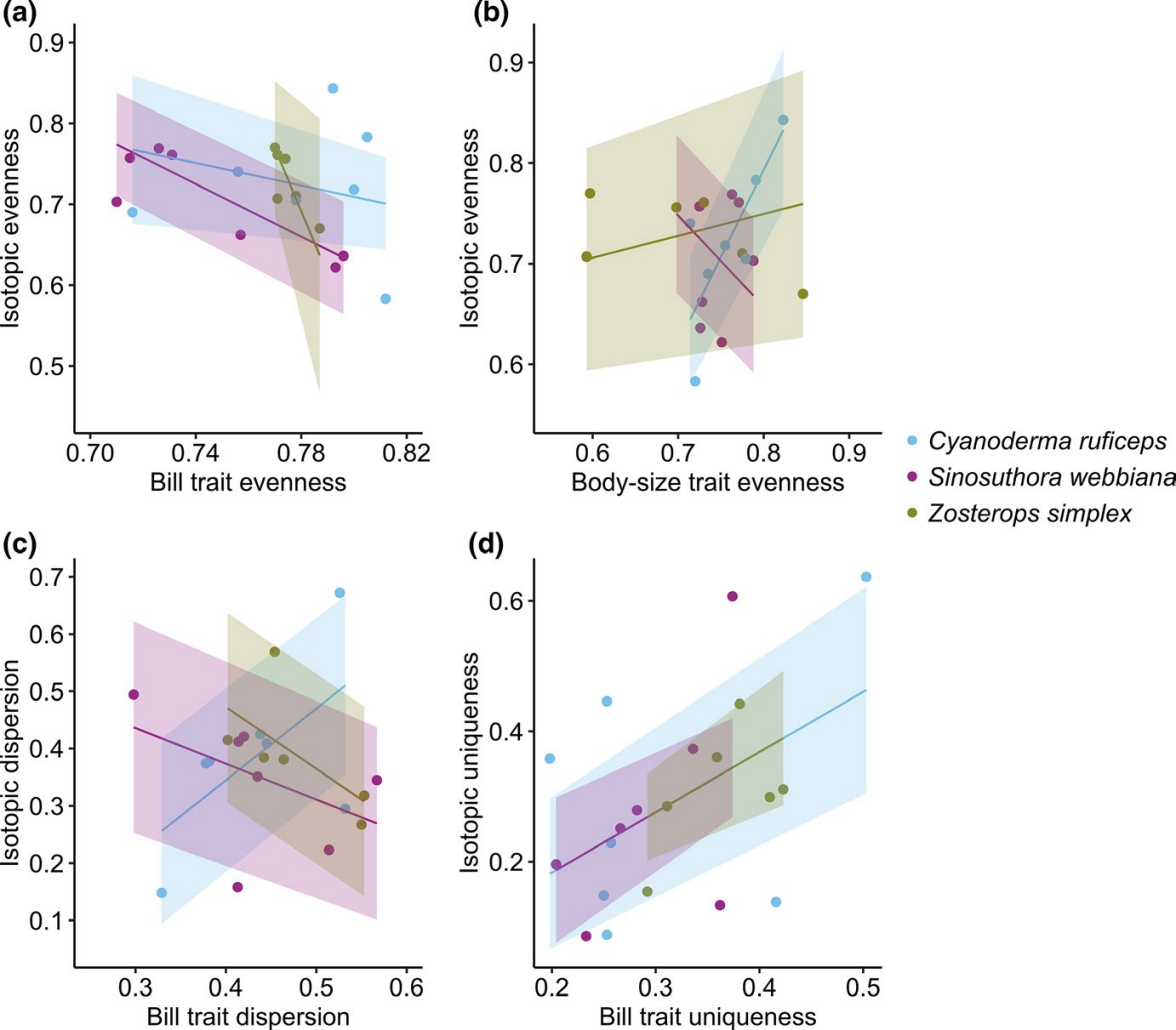
Relationships between isotopic diversity and trait diversity for the passerines. (a) Isotopic evenness versus bill trait evenness; (b) isotopic evenness versus body size trait evenness; (c) isotopic dispersion versus bill trait dispersion; (d) isotopic uniqueness versus bill trait uniqueness. Each dot denotes a population. The solid lines and shaded areas denote the predicted means and their 95% confidence intervals

All three species showed a positive association between isotopic uniqueness and bill trait uniqueness (*F*_1,19_ = 5.95, *p* = 0.02; Table 3 and Figure 3). This means for a population with more individuals occupying unique bill trait positions, more individuals were also occupying unique isotope niche (Figure 3 and Figure S7). For evenness, species × bill and species × body size effects were both significant (species: *F*_2,12_ = 0.38, *p* = 0.69; bill: *F*_1,12_ = 3.70, *p* = 0.08; body size: *F*_1,12_ = 0.23, *p* = 0.64; species × bill: *F*_2,12_ = 3.73, *p* = 0.05; species × body size: *F*_2,12_ = 5.20, *p* = 0.02). For dispersion, species × bill was significant (species: *F*_2,15_ = 0.30, *p* = 0.75; bill: *F*_1,15_ = 0.01, *p* = 0.92; species × bill: *F*_2,15_ = 3.66, *p* = 0.05). None of the effects were significant for divergence (*p* > 0.3). Of the three species, *C. ruficeps* tended to have more positive isotope‐trait association, whereas *S. webbiana* more negative (Figure 3 and Figure S7). However, species‐specific slopes and post hoc comparisons indicated that these species‐specific patterns were generally weak (Table 3).

**TABLE 3:** Parameter estimates and post hoc comparisons for the reduced models of isotopic diversity metrics as functions of species and trait diversity metrics for the passerines

Model	Estimate (95% CI)	Post hoc comparisons
Eveness		
Intercept: Cr	−0.05 (−1.12 to 1.02)	
Intercept: Sw	2.60 (−0.61 to 5.81)	
Intercept: Zs	6.63 (−4.69 to 17.95)	
Slope of bill: Cr	−0.69 (−2.00 to 0.61)	Cr versus Sw: *t* = 3.05, *p* = 0.06
**Slope of bill: Sw**	−1.63 (−4.78 to −0.3)	Cr versus Zs: *t* = 2.32, *p* = 0.23
Slope of bill: Zs	−7.81 (−20.43 to 7.41)	Sw versus Zs: *t* = 0.97, *p* = 1
**Slope of body size: Cr**	1.73 (0.63–2.83)	Cr versus Sw: *t* = 1.10, *p* = 1
Slope of body size: Sw	−0.90 (−3.89 to 2.08)	Cr versus Zs: *t* = 1.11, *p* = 1
Slope of body size: Zs	0.22 (−2.30 to 2.74)	Sw versus Zs: *t* = −1.38, *p* = 1
Dispersion		
Intercept: Cr	−0.16 (−0.72 to 0.41)	
Intercept: Sw	0.62 (−0.7 to 1.95)	
Intercept: Zs	0.90 (−0.68 to 2.49)	
Slope of bill: Cr	1.25 (−0.04 to 2.54)	Cr versus Sw: *t* = 2.32, *p* = 0.11
Slope of bill: Sw	−0.63 (−3.64 to 2.39)	Cr versus Zs: *t* = 2.27, *p* = 0.11
Slope of bill: Zs	−1.08 (−4.55 to 2.39)	Sw versus Zs: *t* = 0.46, *p* = 1
Uniqueness		
Intercept	0.00 (−0.26 to 0.26)	
**Slope of bill**	0.92 (0.13–1.72)	

Note

From the full models, we obtained three sets of reduced models (*Eveness*, *Dispersion*, *Uniqueness*; none of the effects in the full model of *Divergence* were significant) with only the significant main and interaction effects retained. The estimates and their 95% CI were the predicted species‐specific intercepts and slopes for *Evenness* and *Dispersion*, and species‐averaged intercept and slope for *Uniqueness*. The slopes that are significantly different from 0 (95% CI did not overlap 0) are in bold. Post hoc comparisons with Bonferroni adjustment were performed to test species differences in slope. Species names are abbreviated: *Cyanoderma ruficeps* (Cr), *Sinosuthora webbiana* (Sw), *Zosterops* *simplex* (Zs).

### 3.4 Niche variation hypothesis

**FIGURE 4:**
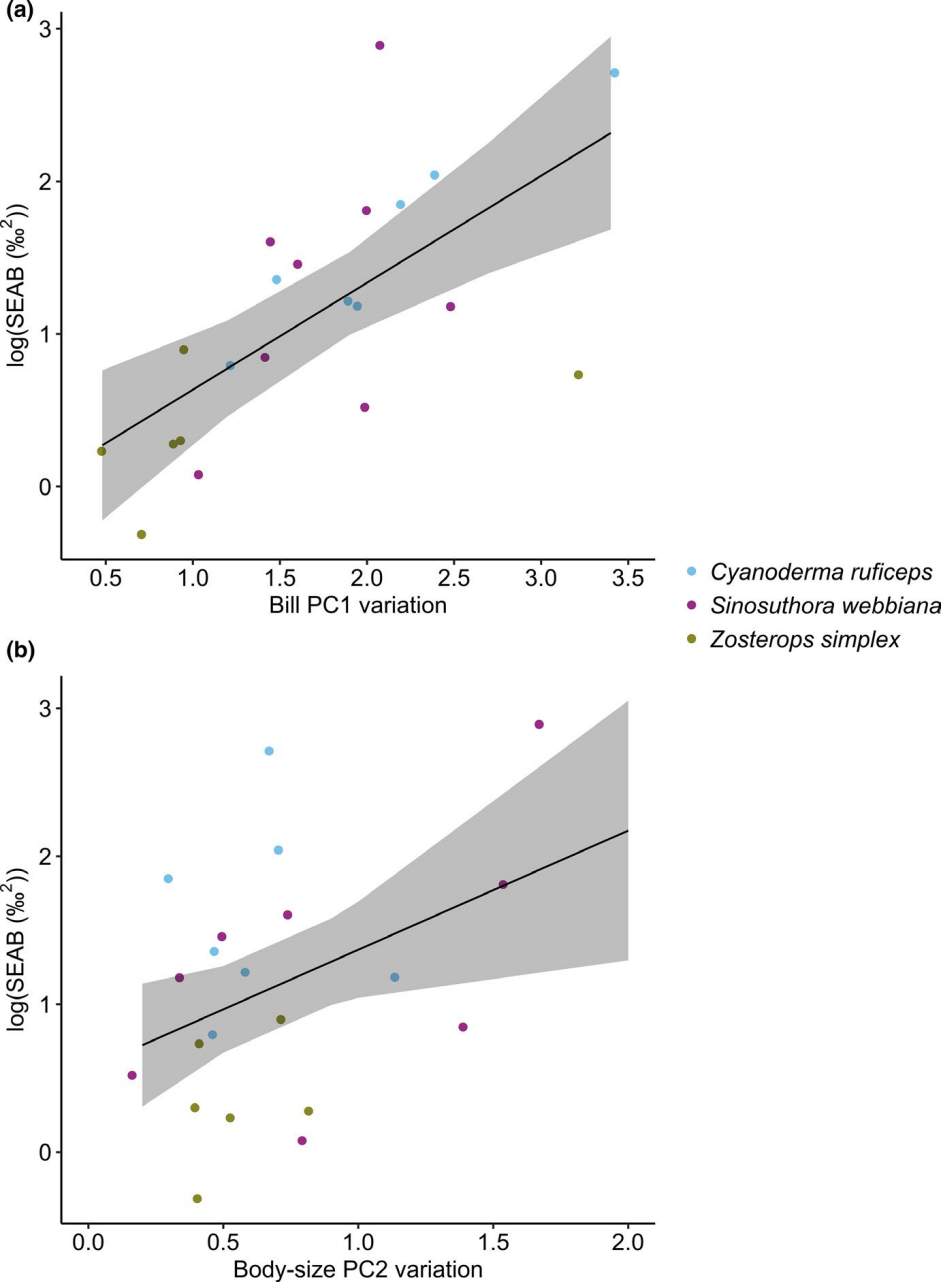
Morphological variation and isotope niche width of the passerines. Bill PC1 variation (a) and body size PC2 variation (b) both increased with isotope niche width (SEAB). Each dot represents a bird population. The solid lines and shaded areas are predicted means and their 95% confidence intervals, based on the reduced model: SEAB = Bill PC1 variation + Body size PC2 variation

The variation of bill PC1 and body size PC2 both increased with isotope niche width (SEAB) across the three species (bill PC1: *F*_1,16.7_ = 18.62, *p* = 0.0005; body size PC2: *F*_1,17.7_ = 6.89, *p* = 0.02; variance explained by random effect “site” and residuals are 0.01 ± 0.1 STD and 0.31 ± 0.56 STD, respectively; Figure 4). The variation of bill PC2 and body size PC1, on the other hand, did not influence isotope niche width. Therefore, the NVH was supported across the three species for isotopic niche width and two of the four traits (bill PC1, body size PC2).

## 4 DISCUSSION

Bird functional traits have been nicely matched to their trophic niche at macro‐evolutionary level (Pigot et al., 2016, 2020). However, at individual and population level, niche–trait relationships are quite complex. In this study, we showed that for all three passerine species, the more unique individual birds were in their bill trait space, the more unique they were in isotope space (Table 3 and Figure 3), indicating IS in both niche and morphology. Furthermore, this individual‐level pattern corresponded to a positive association between bill size variation (variation of bill PC1 primarily reflected variation in the length, width, and depth of bill) and isotope niche width at population level (Figure 4), providing strong evidence for the NVH.

### 4.1 Bill and body size variation

Although body size variation (variation of body size PC2 primarily reflected variation in the lengths of tarsus and wings) also increased with isotope niche width at population level across the three species, the strength of this association was weaker than that between bill size variation and isotope niche width (Figure 4). In fact, when one of the sites with the extremely large isotope niche width was removed (i.e., HEH), body size variation was no longer a significant effect whereas bill size variation remained significant (bill size variation: *F*_1,16_ = 9.77, *p* = 0.007; body size variation: *F*_1,16_ = 1.84, *p* = 0.19). Furthermore, at individual level, only *C. ruficeps* showed a positive association in interindividual differences between body size trait and isotope niche (evenness), whereas *S. webbiana* and *Z. simplex* showed no relationship in interindividual differences between body size trait and isotope niche (Table 3 and Figure 3). Therefore, compared to bill variation, passerine body size variation is likely influenced by more ecological factors than captured by isotope niche.

It is not surprising to find a positive association between bill trait variation and isotope niche width consistently across the species considering that bill is a major feeding apparatus and isotope niche is closely linked to diet. Body size, on the other hand, is critical to many eco‐physiological processes in organisms and likely influenced by many ecological factors. For example, an inverse relationship between body size and ambient temperature (Bergmann's rule) due to thermoregulation constraints has been reported for many passerines (e.g., Gardner et al., 2009; Graves, 1991; Yom‐Tov, 2001). On the other hand, food availability has also been suggested to influence passerine body size, which in some cases outweighed the influence of temperature (e.g., Husby et al., 2011; Yom‐Tov et al., 2006). Because passerine body size has complex relationships with many ecological factors, it is less likely to exhibit the NVH with just one niche dimension (isotope niche). Niche width quantified in multidimensional niche space (e.g., diet, habitat/substrate, food availability, temperature) may be needed to test the NVH between body size and niche width.

### 4.2 Species differences

One of the three species, *S. webbiana*, was shown previously to have increased bill‐shape variation with increased isotope niche width (Hsu et al., 2014). We combined the *S. webbiana* data from Hsu et al. (2014) and newly added data from current study, which re‐confirmed the NVH between bill morphology and isotope niche in this species. Interestingly, *S. webbiana* was the only species that had a negative relationship between isotope diversity and trait diversity at individual level (decreasing bill trait evenness with increasing isotopic evenness; Table 3 and Figure 3), which did not support niche expansion through individual differentiation in morphology. This negative relationship indicated that as individuals were increasingly packed within a small region of bill trait space, they became more evenly distributed in the isotope space. Because *S. webbiana* form social flocks (Severinghaus, 1991), it is possible that they differentiate isotope niche at very fine scale through feeding preference and/or microhabitat use to reduce competition and the need for which could intensify for populations with more packed trait space. The *S. webbiana* flocks are quire flexible, and sometimes, a few individuals might linger in a place while the main flock moved on (Severinghaus, 1991), suggesting fine‐scaled temporal segregation despite being a flock‐forming species.

Contrary to our expectation, the habitat specialist of the three species, *C. ruficeps*, exhibited consistently positive relationships in interindividual differences between trait value and isotope niche (bill uniqueness versus isotopic uniqueness, body size evenness versus isotopic evenness; Table 3 and Figure 3), as well as between isotope niche width and trait variation (Figure 4). Therefore *C. ruficeps* provided an even stronger case for the NVH than *S. webbiana*. On the other hand, *Z. simplex* exhibited weaker NVH compared to the other two species, as expected (Table 3, Figures 3 and 4).

Across the study sites, *Z. simplex* are more restricted in their elevational range (<1,500 m) than the other two species (>2,000 m; Table 1), which could have contributed to their lower trait variation and isotope niche width (Figures 1 and 2; Figures S4–S6). Observations suggest that *Z. simplex* have a more specialized diet, preferring fruits and nectars, whereas *C. ruficeps* and *S. webbiana* have more generalized diets (Chen & Chou, 1999; Severinghaus et al., 2012; Wilman et al., 2014). In this study, we found that *Z. simplex* occupied a slightly lower position along the δ^15^N axis than *C. ruficeps* and *S. webbiana* (Figure 1 and Figure S3), suggesting more plant‐based diets in *Z. simplex*. Species at intermediate trophic levels (omnivores) have been suggested to have a higher likelihood for phenotypic divergence relative to species at other trophic levels (Maldonado et al., 2017; Svanbäck et al., 2015). Therefore, the lack of the NVH in *Z. simplex* could be partly due to their specialized diet at lower trophic level, offering little opportunity for niche shift. This is in contrast with *S. webbiana*, which exhibited a great amount of flexibility in shifting their isotope niche across sites (Figure S3). Finally, compared to *C. ruficeps* and *S. webbiana*, *Z. simplex* are more likely to engage in altitudinal migration between seasons (Y. Hsu, unpublished data); thus, they may be less prone to develop fine niche–trait relationship. Further investigation of niche–trait relationship in *Z. simplex* would benefit from more understanding of their altitudinal migration patterns.

### 4.3 Interindividual differences in isotope space and trait space

The four diversity metrics provide useful tools to quantify how individuals can be specialized in isotope space and trait space with respect to population center (i.e., divergence, dispersion) as well as neighbors (i.e., evenness, uniqueness). The ecological and evolutionary implications of these interindividual differences warrant more studies. For instance, in current study, *S. webbiana* had a positive relationship between bill trait uniqueness and isotopic uniqueness, but also a negative relationship between bill trait evenness and isotopic evenness. Therefore, as the individuals differentiated in their isotope niche, the interindividual differences in their bill morphology may increase or decrease depending on which aspect of their morphological diversity was quantified. Even for populations with individuals packed within a small region of trait space (low bill trait evenness in *S. webbiana*), it is still possible that individuals could occupy relatively unique or very similar trait positions (high or low bill trait uniqueness in *S. webbiana*), creating different interindividual patterns. As more tools become available to quantify interindividual differences, our understanding of the possible links between IS and population variation in niche space and trait space is surely to grow.

## 5 CONCLUSION

This is one of the few empirical studies testing niche–trait relationships at both individual and population level in a multi‐species, multi‐sites system. We found that individual uniqueness in isotope niche space and bill trait space were positively associated across the three passerine species (*C. ruficeps*, *S. webbiana*, and *Z. simplex*). Furthermore, isotope niche width and bill size variation at population level were also positively associated across the species. Therefore, despite some differences in the strength of niche–trait relationships among species, this study provided clear evidence for the NVH between isotope niche width and bill size variation in these passerine species.

## CONFLICTS OF INTEREST

The authors declare that there is no conflict of interest.

## AUTHOR CONTRIBUTIONS

Pei‐Jen L. Shaner: Conceptualization; design of methodology; data analysis; manuscript writing; Yin‐Kai Chen: Design of methodology; data collection; data analysis; Yu‐Cheng Hsu: Design of methodology; data collection; manuscript writing. All authors contributed critically to the drafts and gave final approval for publication.

### OPEN RESEARCH BADGES

This article has earned an Open Data Badge for making publicly available the digitally‐shareable data necessary to reproduce the reported results. The data is available at https://doi.org/10.5061/dryad.j3tx95xd8.

## Supporting information

Supplementary Material

## Data Availability

Data on plant foliar isotope values, bird feather isotope values, and trait values are available from the Dryad Digital Repository https://doi.org/10.5061/dryad.j3tx95xd8.
