# Supplementary material for: Niche–trait relationships at individual and population level in three co‐occurring passerine species
Source: Ecol Evol. 2021 May 2;11(12):7378–89. doi: 10.1002/ece3.7569 (PMC8216981; doi:10.1002/ece3.7569)

**Supporting Information**

**Niche-trait relationships at individual and population level in three co-occurring passerine species**

Pei-Jen Lee, Shaner^1*^, Yin-Kai, Chen ^1^, Yu-Cheng Hsu^2*^

^1^Department of Life Science, National Taiwan Normal University, Taipei, Taiwan

^2^Department of Natural Resources and Environmental Studies, National Dong Hwa University, Hualien, Taiwan

*Corresponding author:

Y. Hsu (ycsheu@gms.ndhu.edu.tw)

**Table S1.** Linear models for technician effect on morphological measurements of *Cyanoderma ruficeps*, *Sinosuthora webbiana* and *Zosterops simplex*. The linear models of the six morphological traits were fitted for each bird species to obtain residual values with technician effect controlled for.

| Trait | Effect | DF | Sum Sq | *F* | *P* | *r*^2^ |
| --- | --- | --- | --- | --- | --- | --- |
| *Cyanoderma ruficeps* | | | | | | |
| Bill length | Technician identify | 8 | 32.01 | 12.99 | <0.0001 | 0.27 |
|  | Residuals | 280 | -- | -- | -- | -- |
| Bill width | Technician identify | 8 | 1.72 | 4.27 | <0.0001 | 0.11 |
|  | Residuals | 280 | -- | -- | -- | -- |
| Bill depth | Technician identify | 8 | 6.23 | 9.94 | <0.0001 | 0.22 |
|  | Residuals | 280 | -- | -- | -- | -- |
| Wing length | Technician identify | 8 | 117.6 | 3.04 | 0.003 | 0.08 |
|  | Residuals | 280 | -- | -- | -- | -- |
| Head length | Technician identify | 8 | 43.67 | 13.24 | <0.0001 | 0.27 |
|  | Residuals | 280 | -- | -- | -- | -- |
| Tarsus length | Technician identify | 8 | 58.23 | 14.29 | <0.0001 | 0.29 |
|  | Residuals | 280 | -- | -- | -- | -- |
| *Sinosuthora webbiana* | | | | | | |
| Bill length | Technician identify | 5 | 17.29 | 23.24 | <0.0001 | 0.31 |
|  | Residuals | 253 | -- | -- | -- | -- |
| Bill width | Technician identify | 5 | 4.79 | 17.37 | <0.0001 | 0.26 |
|  | Residuals | 253 | -- | -- | -- | -- |
| Bill depth | Technician identify | 5 | 6.7 | 15.72 | <0.0001 | 0.24 |
|  | Residuals | 253 | -- | -- | -- | -- |
| Wing length | Technician identify | 5 | 220.43 | 7.87 | <0.0001 | 0.13 |
|  | Residuals | 253 | -- | -- | -- | -- |
| Head length | Technician identify | 5 | 18.28 | 12.86 | <0.0001 | 0.2 |
|  | Residuals | 253 | -- | -- | -- | -- |
| Tarsus length | Technician identify | 5 | 23.69 | 9.17 | <0.0001 | 0.15 |
|  | Residuals | 253 | -- | -- | -- | -- |
| *Zosterops simplex* | | | | | | |
| Bill length | Technician identify | 8 | 10.83 | 8.76 | <0.0001 | 0.34 |
|  | Residuals | 135 | -- | -- | -- | -- |
| Bill width | Technician identify | 8 | 3.82 | 13.03 | <0.0001 | 0.44 |
|  | Residuals | 135 | -- | -- | -- | -- |
| Bill depth | Technician identify | 8 | 3.64 | 14.76 | <0.0001 | 0.47 |
|  | Residuals | 135 | -- | -- | -- | -- |
| Wing length | Technician identify | 8 | 62.59 | 2.58 | 0.001 | 0.13 |
|  | Residuals | 135 | -- | -- | -- | -- |
| Head length | Technician identify | 8 | 31.52 | 13.31 | <0.0001 | 0.44 |
|  | Residuals | 135 | -- | -- | -- | -- |
| Tarsus length | Technician identify | 8 | 54.79 | 22.98 | <0.0001 | 0.58 |
|  | Residuals | 135 | -- | -- | -- | -- |

**Table S2.** Linear models for technician and species effect on morphological measurements of *Cyanoderma ruficeps*, *Sinosuthora webbiana* and *Zosterops simplex*. The linear models of the six morphological traits were based on the data pooled across the bird species to examine whether technician effect varies with species.

| Trait | Effect | DF | Sum Sq | *F* | *P* |
| --- | --- | --- | --- | --- | --- |
| Bill length | Technician identify | 9 | 325.44 | 166.84 | <0.0001 |
|  | Species | 2 | 2066.66 | 4767.59 | <0.0001 |
|  | Technician identity × species | 12 | 12.84 | 4.94 | <0.0001 |
|  | Residuals | 668 | 144.78 |  |  |
| Bill width | Technician identify | 9 | 20.88 | 46.93 | <0.0001 |
|  | Species | 2 | 190.87 | 1930.61 | <0.0001 |
|  | Technician identity × species | 12 | 3.23 | 5.45 | <0.0001 |
|  | Residuals | 668 | 33.02 |  |  |
| Bill depth | Technician identify | 9 | 127.64 | 198.74 | <0.0001 |
|  | Species | 2 | 763.02 | 5346.14 | <0.0001 |
|  | Technician identity × species | 12 | 4.64 | 5.41 | <0.0001 |
|  | Residuals | 668 | 47.67 |  |  |
| Wing length | Technician identify | 9 | 285.32 | 6.66 | <0.0001 |
|  | Species | 2 | 536.62 | 56.34 | <0.0001 |
|  | Technician identity × species | 12 | 72.65 | 1.27 | <0.0001 |
|  | Residuals | 668 | 3181.43 |  |  |
| Head length | Technician identify | 9 | 70.34 | 22.97 | <0.0001 |
|  | Species | 2 | 353.55 | 519.47 | <0.0001 |
|  | Technician identity × species | 12 | 25.40 | 6.22 | <0.0001 |
|  | Residuals | 668 | 227.32 |  |  |
| Tarsus length | Technician identify | 9 | 214.05 | 50.66 | <0.0001 |
|  | Species | 2 | 2410.64 | 2567.55 | <0.0001 |
|  | Technician identity × species | 12 | 16.62 | 2.95 | 0.0005 |
|  | Residuals | 668 | 313.59 |  |  |

**Fig. S1.** Photos of the study sites taken at the bird-netting locations. For descriptions of the sites, see Table 1.


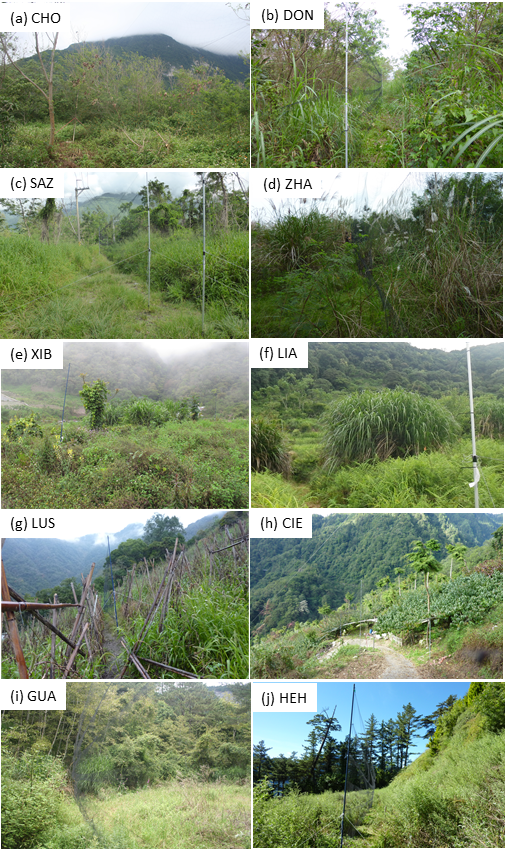


**Fig. S2.** Photos of passerine morphology. (a) bill length measured from tip to skull along the culmen, (b) bill width measured at the proximal edges of the nostril, (c) bill depth measured at the proximal edge of the nostril, (d) head length measured from the tip of the bill to the back of the head then subtracting bill length, (e) tarsus length measured from the inner bend of the tibiotarsal articulation to the base of the toes, (f) wing length measured from carpal joint to wingtip. The species is *Zosterops simplex*.


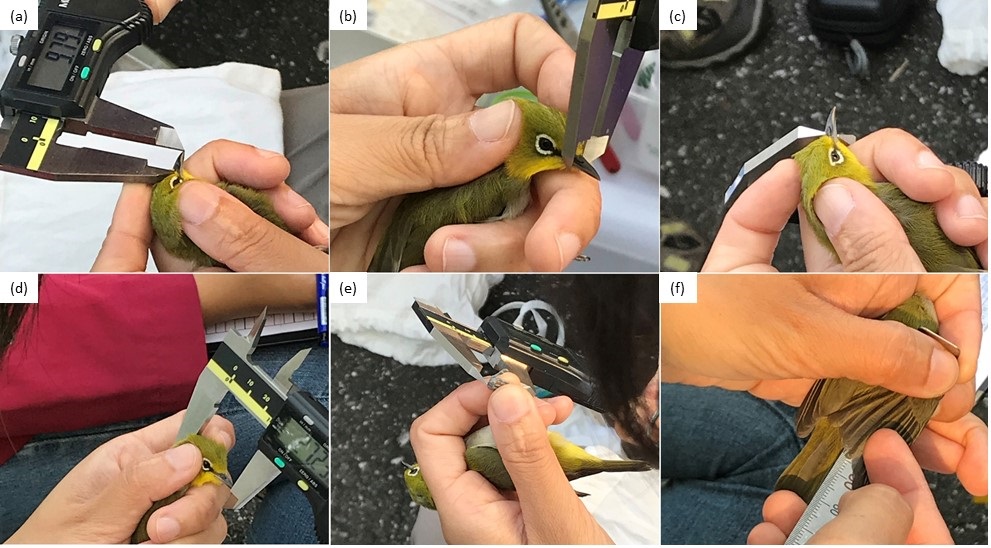


**Fig. S3.** Site-specific isotope data of the passerines and plants. The feather isotope values are unadjusted for the baseline (plant foliar) values. The filled circles are mean values and error bars denote ± 1 standard deviation. Grey: plants; orange: *Cyanoderma ruficeps*; pink: *Sinosuthora webbiana*; green: *Zosterops simplex*. For descriptions of the sites, see Table 1.


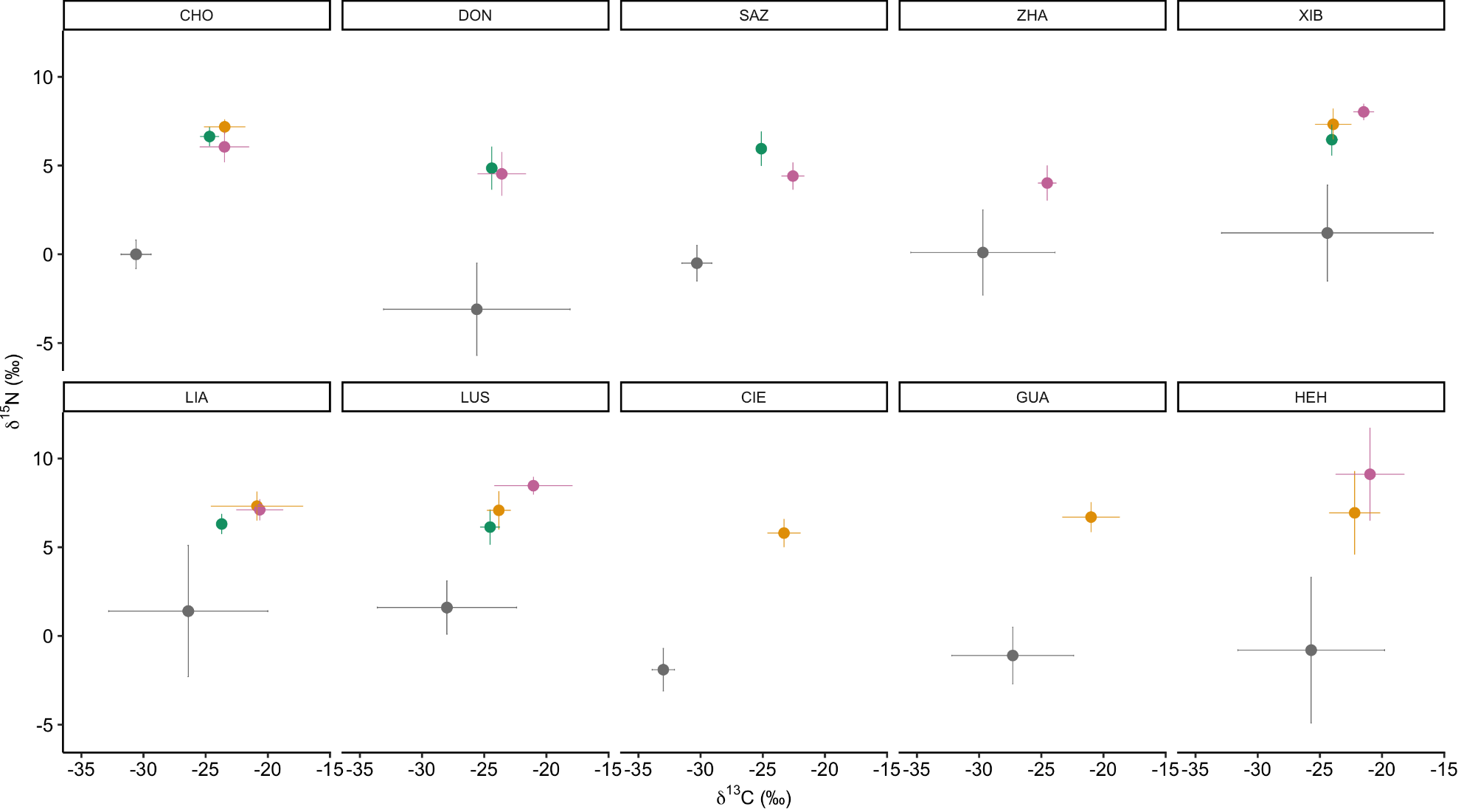


**Fig. S4.** Bill and size morphology of the passerines. The trait values of *Cyanoderma ruficeps* (Cr), *Sinosuthora webbiana* (Sw), and *Zosterops simplex* (Zs) were the residuals from the linear models that accounted for the technician effect (Table S1). Each boxplot shows the median (the horizontal line), 25^th^-75^th^ percentiles (the box), and 2.5^th^-97.5^th^ (the whiskers) percentiles of the residual trait values.


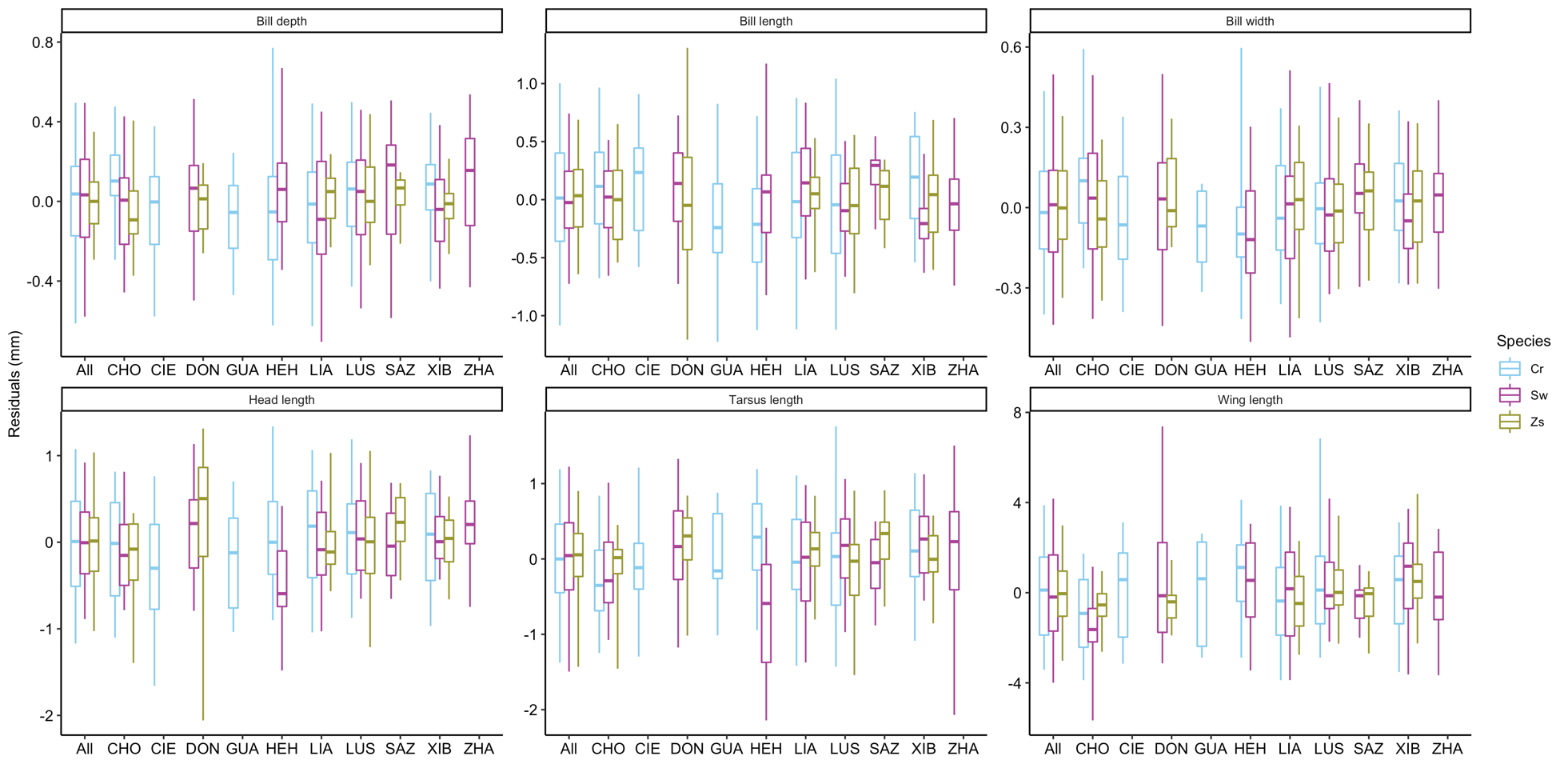


**Fig. S5.** Principal component scores of bill and body-size traits of the passerines. The PC scores of *Cyanoderma ruficeps* (Cr), *Sinosuthora webbiana* (Sw), and *Zosterops simplex* (Zs) were based on the residuals from the linear models that accounted for the technician effect (Table S1). Higher bill PC1 scores indicated larger bills (longer, wider and thicker bills), and higher bill PC2 scores slender bills (long and narrow bills; Table 2). Higher body-size PC1 scores indicated larger size (longer heads, tarsus and wings), and higher body-size PC2 scores longer tarsus with shorter wings; Table 2). Each boxplot shows the median (the horizontal line), 25^th^-75^th^ percentiles (the box), and 2.5^th^-97.5^th^ (the whiskers) percentiles of the PC scores.


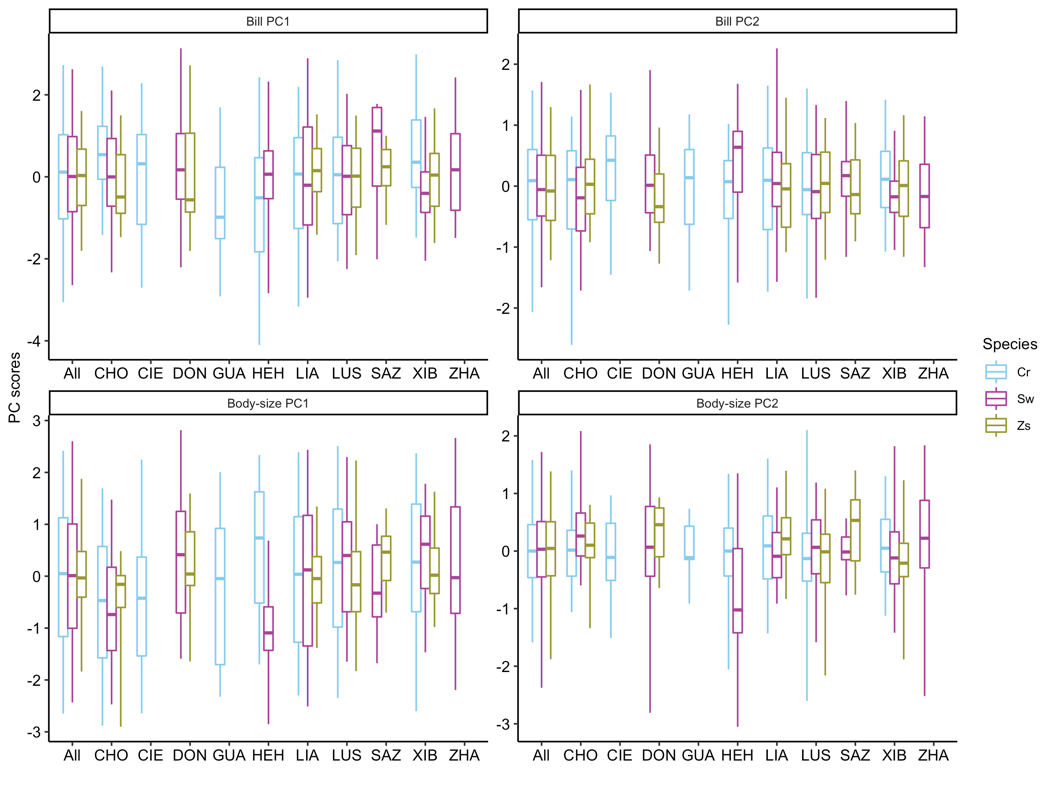


**Fig. S6.** Population-level isotope niche of the passerines. Adjusted isotope values of individual birds (δ^13^C_adj_ and δ^15^N_adj_) were used to indicate individuals’ niche positions. Bayesian standardized ellipse area (SEAB) was used to estimate population-level niche width based on individuals’ isotope values. Each unfilled circle represents the isotope values of a unique individual, and the ellipses are 10 posterior draws of the 4,000 Bayesian standard ellipses to illustrate population-level niche width. The panels are arranged by species from left to right (Cr: *Cyanoderma ruficeps*, Sw: *Sinosuthora webbiana*, Zs: *Zosterops simplex*), and by sites from top to bottom (CHO, DON, SAZ, ZHA, XIB, LIA, LUS, CIE, GUA, HEH). The empty space indicates that a species did not occur at the site.


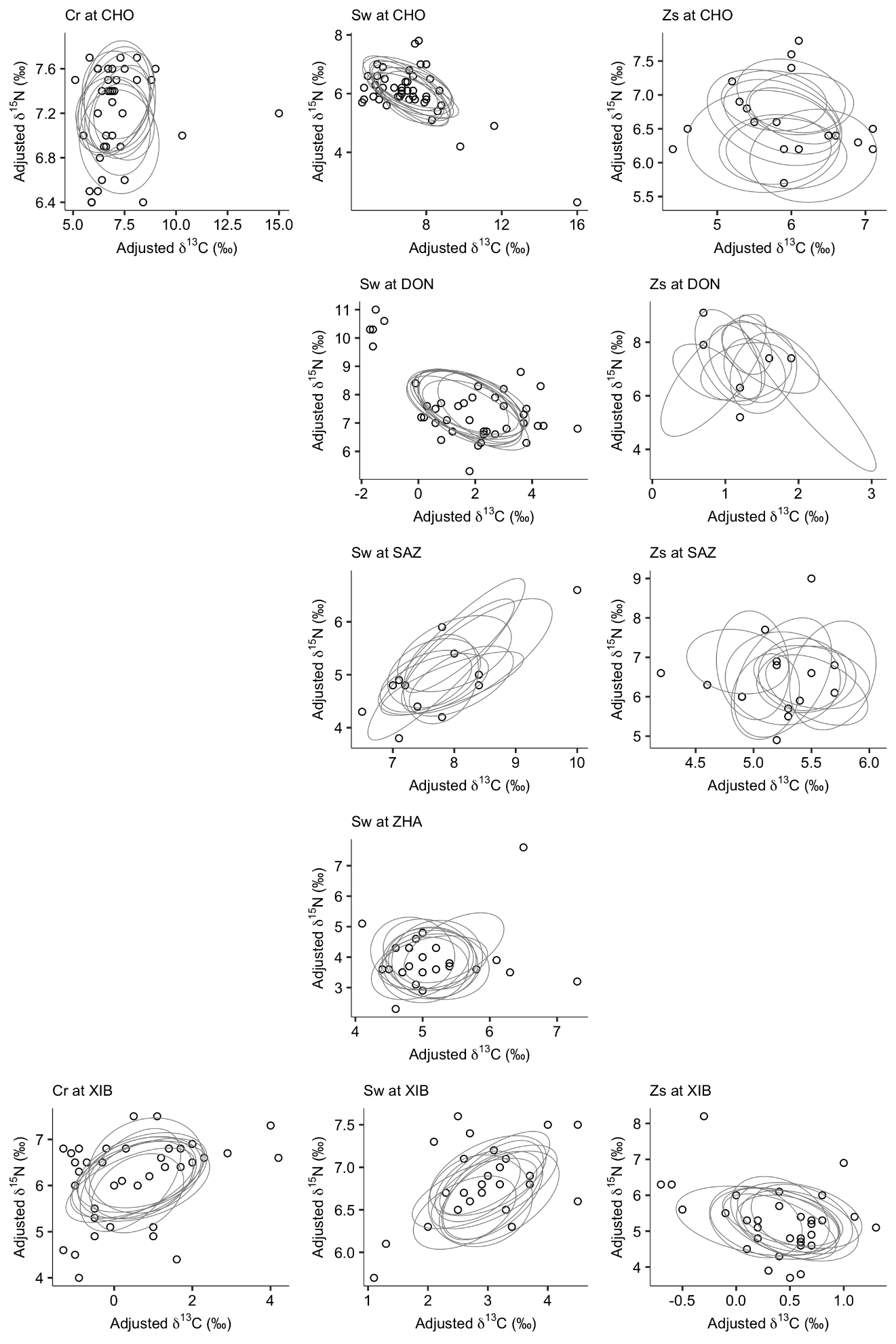


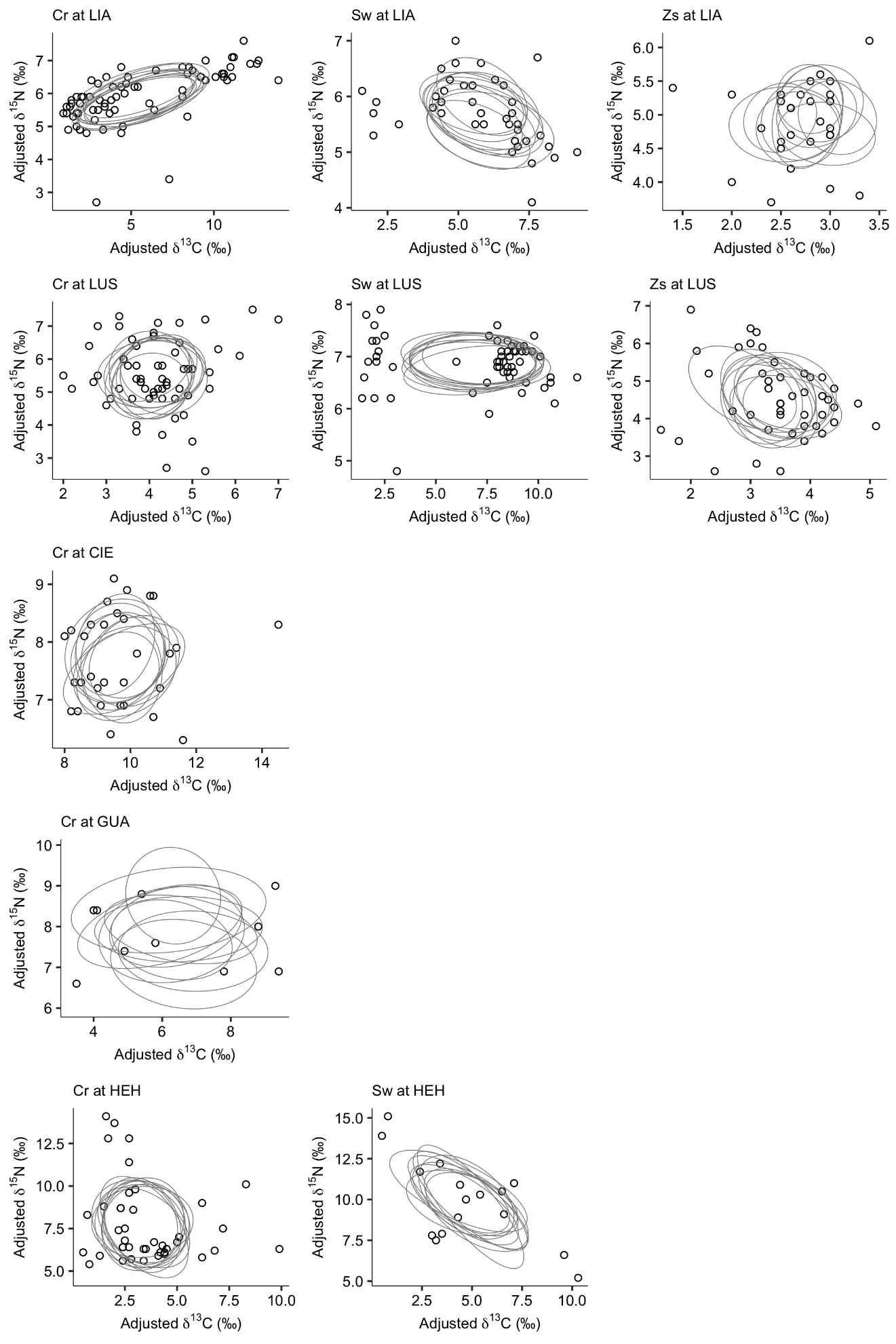


**Fig. S7.** Examples of isotopic and trait diversity metrics for the passerines. (a) Body-size trait and isotopic evenness for *Cyanoderma ruficeps* populations with the lowest, median and highest (top to bottom) isotopic evenness; (b) Bill trait and isotopic evenness for *Sinosuthora webbiana* populations with the lowest, median and highest (top to bottom) isotopic evenness; (c) Bill trait and isotopic uniqueness for bird populations with the lowest, median and highest (top to bottom) isotopic uniqueness. Evenness is the regularity of points (position and importance) along the shortest tree linking all the points (green dendrogram). Uniqueness is measured as the weighted-mean of distances to nearest neighbour (black arrows). For r scripts to draw these figures, the reader is referred to Cucherousseta & Villéger (2015).

(a)


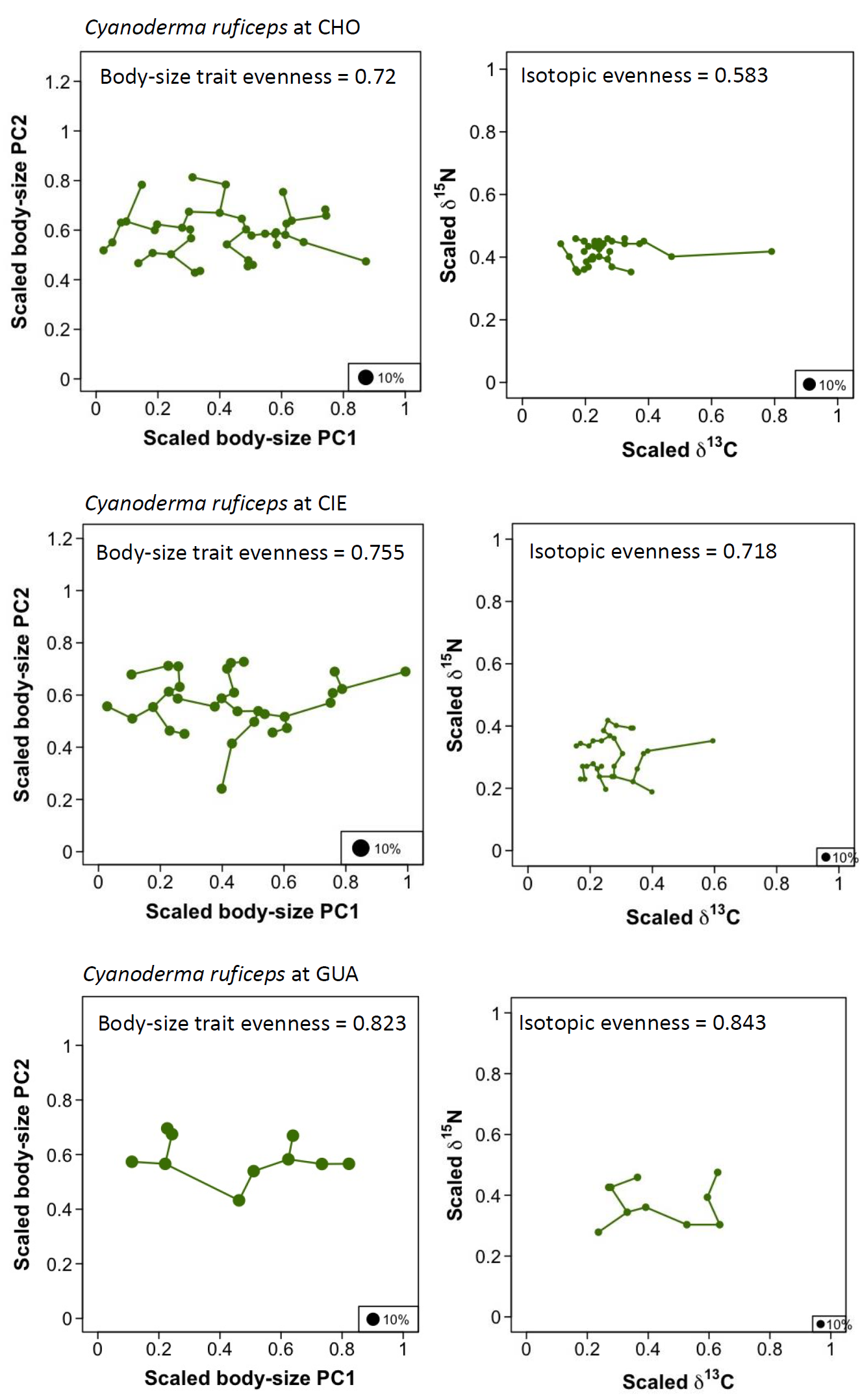


(b)


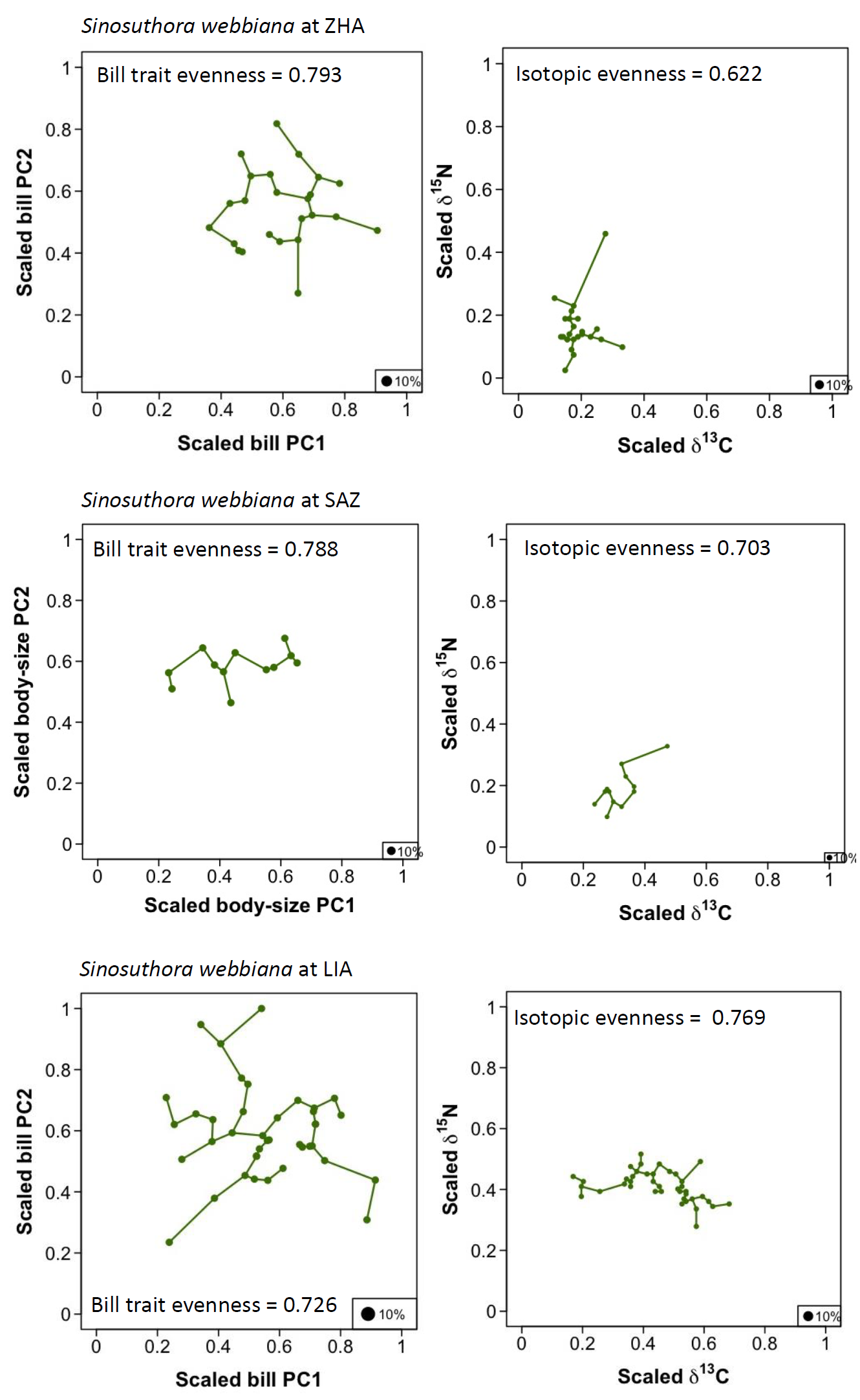


(c)


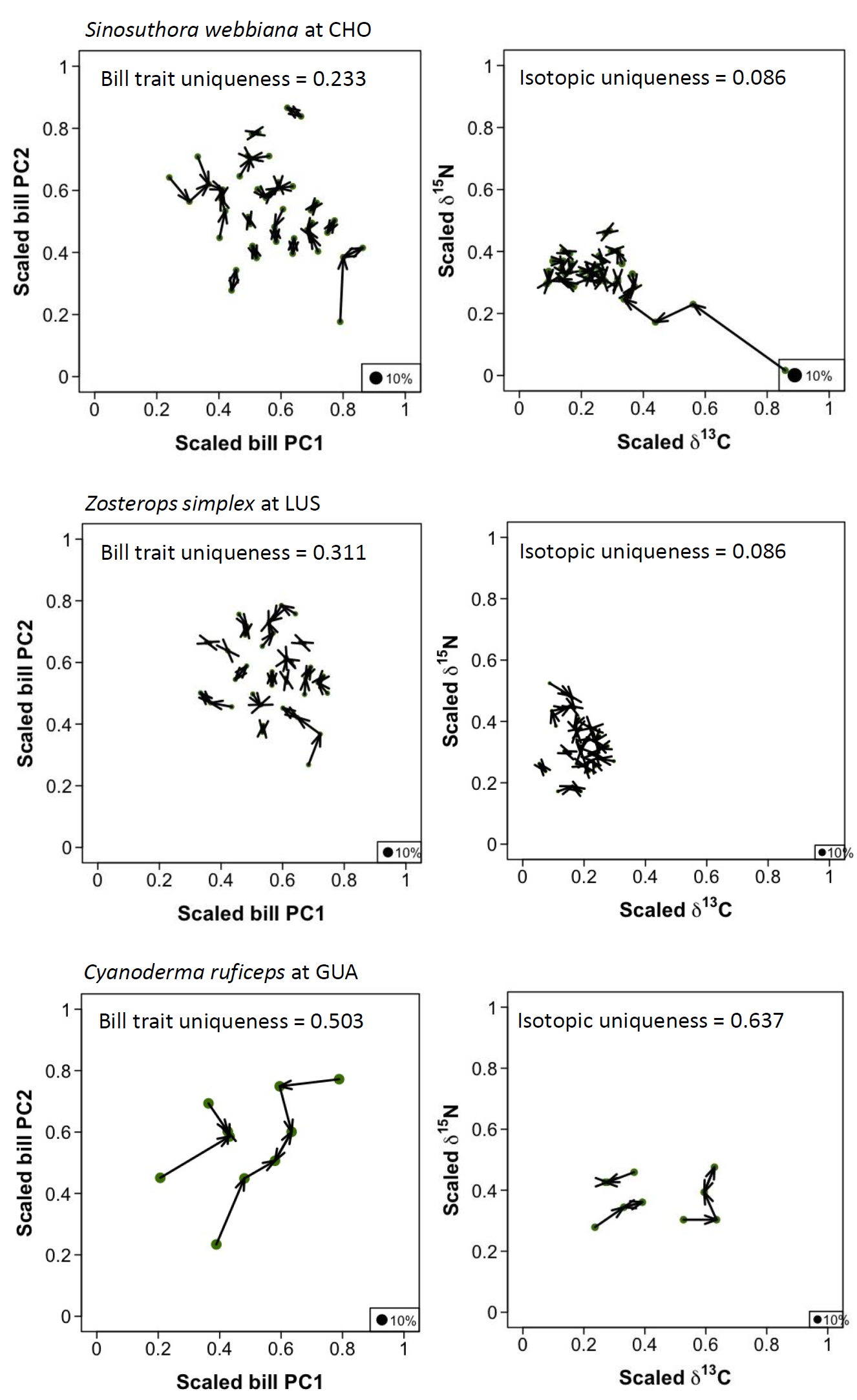

Supplement: Supplementary file 1 — Supplementary Material [file ECE3-11-7378-s001.docx]
